# Combining Fungicides and Prospective NPR1-Based “Just-in-Time” Immunomodulating Chemistries for Crop Protection

**DOI:** 10.3389/fpls.2017.01715

**Published:** 2017-10-04

**Authors:** Xiahezi Kuai, Charles Barraco, Charles Després

**Affiliations:** Department of Biological Sciences, Brock University, St. Catharines, ON, Canada

**Keywords:** crop protection, pendulum, agriculture, agrochemical, fungicide, immunomodulator, differential scanning fluorimetry, fungicide resistance

## Abstract

Each year, crop yield is lost to weeds competing for resources, insect herbivory and diseases caused by pathogens. To thwart these insults and preserve yield security and a high quality of traits, conventional agriculture makes use of improved cultivars combined with fertilizer and agrochemical applications. However, given that regulatory bodies and consumers are demanding environmentally safer agrochemicals, while at the same time resistance to agrochemicals is mounting, it is crucial to adopt a “holistic” approach to agriculture by not excluding any number of management tools at our disposal. One such tool includes chemicals that stimulate plant immunity. The development of this particular type of alternative crop protection strategy has been of great interest to us. We have approached this paradigm by studying plant immunity, specifically systemic acquired resistance (SAR). The deployment of SAR immunity requires the production by the crop plant of an endogenous small molecule metabolite called salicylic acid (SA). Furthermore, immunity can only be deployed if SA can bind to its receptor and activate the genes responsible for the SAR program. The key receptor for SAR is a transcription coactivator called NPR1. Since discovering this NPR1-SA receptor–ligand pair, we have embarked on a journey to develop novel chemistries capable of deploying SAR in the field. The journey begins with the development of a scalable assay to identify these novel chemistries. One such assay, presented here, is based on differential scanning fluorimetry technology and demonstrates that NPR1 is destabilized by binding to SA.

## Introduction

**FIGURE 1:**
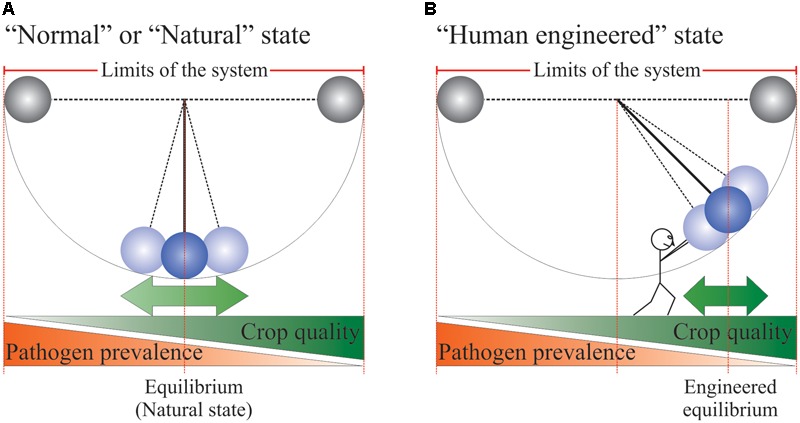
Pendulum analogy of agricultural systems. See text for more details. The little human represents all the non-natural inputs and technologies used to ensure crop yield and quality. **(A)** “Normal or Natural state” of an agricultural system. **(B)** “Human engineered state” of an agricultural system.

A compelling analogy for agricultural systems is that of the simple gravity pendulum (**Figure 1**). Prior to the domestication of plants and any major human interventions on the planet, plant–pathogen interactions in the natural environment would have been at equilibrium or what could be described as the “normal" state. In some years or under certain conditions, the pathogen population may be more prevalent, while in some other instances, it is the host population that would have been more prevalent. These swings were likely never far from equilibrium since the goal of the pathogen population is to “nibble" at the host just enough to ensure that its reproductive cycle is completed, without wiping the host population out of existence. For the host population, its goal is to expend just enough energy to fight the pathogen so that it can complete its own life cycle and set seeds for the next generation. Under this paradigm, a plant population can allow a substantial amount of “yield” loss to pathogen attack as long as the capacity to reproduce is met. Furthermore, along with yield loss, pathogen infections often produce toxic chemicals incompatible with human consumption. This paradigm clearly clashes with human needs and as such, we have invented our agriculture with the goal of moving and holding the pendulum away from pathogen prevalence to maximize plant yield and quality. In human terms, moving and holding the pendulum high above our head requires a constant expense of energy. In agricultural terms, holding the pendulum is achieved through proper agricultural practices, the usage of elite cultivars and the treatment of crops with fertilizers and chemicals such as pesticides. Removal of the forces holding the pendulum would quickly lead to the system reverting to equilibrium or “normality.”

In the developed world, food is plentiful, relatively inexpensive and of high quality. Complacency has set in and made us forget that such abundance does not represent “normality” within the context of human history. In fact, scarcity is the norm and the profusion of high quality food is exceptional and a relatively recent phenomenon, which still has not reached all corners of our planet (Godfray et al., 2010). Since the dawn of agriculture, humans have been battling the elements and fighting crop diseases and weed infestations to safeguard their crops and ensure their own survival. Crop protection is a serious matter and is a constant battle against the tremendous force exerted by a system wanting to return to its equilibrium point, its “normality.” Fungal and bacterial pathogens represent part of this formidable force and are therefore one of the major causes of crop loss worldwide (Oerke and Dehne, 2004). Despite the sophisticated crop protection practices of today, the average yield lost to fungal and bacterial pathogens still hovers around 11% for barley, cottonseed, maize, oilseed rape, potatoes, rice, soybean, cotton, sugar beet, tomatoes, and wheat (numbers from 2001 to 2003; Oerke, 2006). All means that can assist in keeping the pendulum high and away from its natural equilibrium point should be evaluated and potentially developed. In this perspective, we will discuss current countermeasures used to preserve crop yield security and quality threatened by pathogens, specifically fungi. We will also discuss the development of novel crop protection chemistries modulating plant immunity centered on the identification of new agonists of the salicylic acid (SA) receptor NPR1.

### Fungicides: A Key Turning Point in the War against Pathogenic Crop Loss

The application, on a cultivated field, of crop protectants, which are generally grouped under the umbrella-term “pesticides,” plays a significant role in safeguarding crop productivity. When the pathogens to combat are fungi, special pesticides termed fungicides are applied to the crop. Fungicides are chemical agents that, by definition, kill fungi or fungal spores (Haverkate et al., 1969). Humans have been using natural anti-fungal agents to control plants disease long before they realized that fungi were a causative agent of agricultural damage. Dating back to around 60 AD, wine was already used in cereal seed treatment to prevent disease (Russell, 2005). Interests in controlling fungal disease rose greatly after the devastating Irish Potato Famine of the mid-nineteenth century, which resulted in the death of approximately one million people (Gráda and Eiríksson, 2006). The Famine was caused by a fungal disease, called potato late blight. In 1885, the Bordeaux mixture, the first fungicide to be widely used worldwide, was serendipitously discovered by French botanist, Pierre-Marie-Alexis Millardet (Millardet et al., 1933). The Bordeaux mixture, a bitter tasting concoction prepared by mixing copper sulfate (CuSO_4_) and slaked lime [Ca(OH)_2_], was initially used on vines near roadways in order to discourage thieves from stealing grapes. Millardet discovered that downy mildew was less abundant on vines treated with the mixture. This accidental discovery turned out to be a very effective means to control many other diseases and for example, a 20 years study conducted in Vermont, US (1890–1910) showed that the control of potato blight with Bordeaux mixture increased the average yield of the potato crop by 64% (Jones et al., 1912). Up to the early 1900s, agricultural fungicides were mainly homemade by farmers using active ingredients such as sulfur, lime and copper sulfate. However, the 1930s brought a radical change to agriculture with the development of the first synthetic chemical fungicides (nabam, thiram, and zineb) (Gianessi and Reigner, 2005). Yields from potato crops treated with zineb and nabam were 23–35% higher than those obtained by spraying with the Bordeaux mixture (Muncie and Morofsky, 1947; Wilson and Sleesman, 1951). Because of the superior performance of synthetic fungicides, fungicide production rapidly shifted from “do-it-yourself” concoctions to commercially produced chemistries. This paradigm shift signaled the beginning of the crop protection industry as we know it today. To date, some 110 different groups of fungicide with at least 10 different modes-of-actions have been discovered (Source: Fungicide Resistance Action Committee). In modern agriculture, synthetic fungicides, without doubt, play a significant role in safeguarding crop quality and yield. Typically, a synthetic fungicide recommended for a specific crop-fungus couple will provide 90% or greater control of the target disease (Gianessi and Reigner, 2005). Without their protection, it is estimated that the yield of most fruit and vegetable crops would fall by 50–95% due to plant disease (Gianessi and Reigner, 2005).

Unfortunately, despite the effectiveness of fungicide for yield security and the production of toxin-free crops, pathogens respond to the use of these chemicals by developing resistance. Fungicide resistance is said to have occurred when a chemistry with a specific mode-of-action has lost its ability to kill or inhibit fungal growth (Brent and Hollomon, 1995). This natural phenomenon is akin to antibiotics resistance in the treatment of human diseases of bacterial origin. Not unlike the population of the human species, the population of a specific fungal species consists of genetically diverse individuals. Within a fungal population, there are very rare individuals that, by chance, have just the right genetic make-up to survive the normally lethal effects of a fungicide. These are said to be individuals resistant to the fungicide. When a fungicide is used for many seasons to protect a crop, the descendants of these rare resistant individuals are the only ones that can survive the treatment and as a result become the more dominant constituents of the population. As the percentage of resistant fungal individuals increases in the population, the effectiveness of the fungicide decreases to the point where it becomes completely ineffective and/or not economically viable to use as a crop protectant. It is also observed that the more effective a fungicide is at killing the targeted fungus, the higher the selective pressure is on the fungal population and the faster the fungicide resistance develops (Brent and Hollomon, 1995). The first instance of fungicide resistance in the field was observed in 1960 (Eckert, 1982). Up to the 1970s, there were only a few severe cases of fungicide resistance and the time taken for resistance to emerge was relatively long, ranging from 10 to 40 years. However, since the 1970s, the incidence of resistance has increased dramatically. Today, all major groups of fungicides have a reduced breadth of efficacy due to severe cases of resistance in certain fungal populations (Brent and Hollomon, 1995). Furthermore, the elapsed time before resistance emerges is often rather short (under 10 years). For example, in the case of the strobilurin [aka Quinone outside Inhibitors (QoIs)] class of fungicides, which is profusely used because of its activity against all major fungal genera (Heaney et al., 2000), the first case of resistance developed after only 2 years following commercial introduction of the product. The emergence of fungal pathogen resistance to fungicide has become a widespread and severe problem in agriculture, which threatens yield security and crop quality. Alarmingly, food production is not the only way fungicide resistance affects humans. Resistant fungal species are also threatening human health, with the root-cause potentially stemming from agricultural practices.

Typically, when a human is afflicted by a disease, metabolic or pathogenic in origin, it is likely that this person will take medication, which could be, for example, delivered in the form of a pill (oral administration) or intravenously. These routes of administration are designed to provide a specific and precise dosage (an amount of drug/kg body weight) and to confine the drug to the individual afflicted by the disease, without the drug reaching, for example, family members living under the same roof. Contrary to the treatment of human disease, the principal method of fungicide delivery in agriculture is through spraying (Brent and Hollomon, 1995). This application method provides a specific and precise dosage (an amount of fungicide/surface area of cultivated field). However, it does not confine the “medication” only to individuals, but it also “treats” the space between them. As such, spraying requires significantly higher amounts of “medication” for similar therapeutic effects as would be observed in humans. Critically, certain fungicide classes, like Azoles, are utilized to combat both human and crop fungal infections, increasing the probability for resistance to develop. It is thought that the massive use of azoles in agriculture has resulted in the emergence of multi-azole-resistant *Aspergillus fumigatus* isolates, the fungal agent responsible for invasive aspergillosis in human (Verweij et al., 2009). The group also pointed out that in 2004, the volume of azoles and azole-like agricultural fungicides used in the Netherlands was about 320-times higher than that of azoles used in clinical medicine in the vicinity of 130000 kg vs. 400 kg. The large concentrations of applied azoles in agriculture, more than likely, lead to the accelerated evolution of *A. fumigatus* species for azole-resistance giving rise to aspergillosis in humans (Verweij et al., 2009). Such examples of fungicide resistance give ample reasons to look for means to minimize the use of fungicides and embrace a more “holistic” approach to crop protection. The bob of the pendulum is very heavy and recruiting more people to hold it above head reduces the individual effort required to maintain the status quo. The corollary is that under this paradigm, the status quo is not jeopardized should one individual pass away. The pendulum analogy stresses that it is riskier to rely heavily on a single strategy, fungicides, as the pillar of fungal crop protection. Catastrophic crop failures could arise, should certain fungicide classes die on us as a consequence of being ineffective due to fungal resistance.

Despite the large number of fungicide chemical structures developed for agriculture, the target disease in each major crop-fungus couple can only be controlled by three or four different classes of fungicides (Brent and Hollomon, 1995). Current fungicide resistance management strategies include applying a mixture of fungicides with different modes-of-action and following strict guidelines for application regimen and concentrations. In parallel, efforts are made to discover new fungicides with multi-site mode-of-actions (Brent and Hollomon, 1995). These agrochemical management strategies cannot prevent the emergence of fungicide resistance, especially not those relating to human diseases, but they have been shown to be effective at delaying fungal resistance relating to crops.

### “Just-in-Time” Immunomodulating Chemistries for Crop Protection

In human medicine, the use of drugs that kill pathogens, such as fungicides and antibiotics, is not the only solution available to combat microbes. Alternative approaches that rely on boosting the immune system, such as immunization, are an important line of defense against pathogens. The immune systems of plants and animals operate quite differently, but it is nevertheless possible to develop strategies that can boost plant immunity. However, in plants there exist a tradeoff between immunity and growth/development and many attempts at engineering constitutive immunity results in plants with suboptimal growth and development profiles, which affects yield directly (Heil and Baldwin, 2002). This tradeoff can be mitigated to a large extent by inducing immunity at the appropriate time, which can be accomplished by treatments with chemicals that boost immunity on demand and only when needed. This “just-in-time” philosophy was pioneered by Toyota for their Production System. Supplying “what is needed, when it is needed, and in the amount needed” according to their production plan is an important reason for Toyota’s success in the fierce automobile manufacturing market. For Toyota, the “just-in-time” strategy can “eliminate waste, inconsistencies, and unreasonable requirements, resulting in improved productivity.” By analogy, for crops, triggering immunity “just-in-time” would allow for optimum allocation of limited resources to resistance, when needed and for the amount of time needed, and resumption of growth and development once a threat has been neutralized, minimizing yield loss. As an added benefit, since these immunomodulators will target the crop and not the pathogen, the rate at which resistance manifests itself should be virtually nil, if properly implemented. Furthermore, immunomodulators that target NPR1, in principle, could be used across multiple plant species as NPR1 is found and is conserved in all crops of commercial significance (Kuai and Després, 2016). Recent advances in our understanding of the molecular mechanisms of plant immune responses, specifically those regulated by NPR1, have provided us with an opportunity to develop new agrochemicals through target-based pharmaceutical-style approaches, which we will discuss in the following sections.

### The SA-Receptor NPR1 as a Validated Immunomodulating Target

Plants have evolved a variety of perception systems to recognize initial attacks from pathogens (Thomma et al., 2011). These systems are often referred to as microbial- or pathogen-associated molecular patterns (MAMPs or PAMPs)-triggered immunity and effector triggered immunity (Jones and Dangl, 2006). After perceiving the initial threat, plants establish a broad-spectrum and long-lasting resistance, coined systemic acquired resistance (SAR), to protect themselves from the pathogenic invaders (Ryals et al., 1996). Another type of resistance, induced by beneficial microbes, termed Induced Systemic Resistance (ISR), also exists and is reviewed elsewhere (Pieterse et al., 2014). SAR can be induced by avirulent pathogens and can confer resistance to a wide range of normally virulent pathogens, including bacterial, viral, and fungal pathogens. This SAR response is mediated by the phytohormone, SA (Vlot et al., 2009). SA controls plant defense responses through its receptor protein, NPR1. Upon binding with SA, NPR1 undergoes a conformational change allowing it to act as a transcriptional co-activator and to activate the transcription of pathogenesis-related genes, which play a role in plant immunity (Ward et al., 1991; Wu et al., 2012). Although several hormones are involved in modulating SAR (Pieterse et al., 2009), the presence of SA and the NPR1 protein are two absolute requirements for the induction of SAR in plants. Mutant plants that cannot accumulate normal concentration of SA, or with mutations in *NPR1* fail to establish SAR in response to pathogen challenge (Lawton et al., 1995; Cao et al., 1997). Exogenous application of SA can replace an initial avirulent pathogen challenge and stimulate a plant’s innate immune response (White, 1979), which brought to light the feasibility of using chemicals to activate plant immunity on demand. Although SA could, in theory, be used directly as an agrochemical, its application is limited by its chemical stability and rapid catabolic inactivation (Uknes et al., 1993).

The purpose of developing NPR1 agonists, that could deliver a “just-in-time” boost to crop immune system, is not meant to replace the current effective fungicides. The objective is to complement the use of fungicides in a comprehensive anti-resistance strategy to extend their commercial life and to ensure long-term global food security. Since SAR can provide broad-spectrum resistance, prospective NPR1 agonists could also prove effective in controlling bacterial and viral pathogens.

The chemical stimulation of crop immune responses is already successful in controlling pathogens. Benzo (1,2,3)-thiadiazole-7-carbothiolic acid (BTH) was first discovered as an inducer of SAR in 1996 and later brought to market by Syngenta as the active ingredient in a commercial product, Actigard (Bion in Europe). BTH was believed to act at the site or downstream of SA induced defense response pathway because BTH can induce resistance in a mutant plant that cannot accumulate normal concentrations of SA (Lawton et al., 1996). In Wu et al. (2012), we have demonstrated that BTH interacts with the SA-receptor, NPR1, with similar or a slightly better binding affinity than SA. Another example is Bayer CropScience’s recently introduced Isotianil, which protects rice crops from fungal infection by inducing SAR (Ogawa et al., 2011). However, these agrochemicals suffer from limited crop range. It is true that BTH can be used on 122 crop plants in the United States (Syngenta United States label on Actigard 50WG), dosage and registered crops vary by country. For example, in Canada, Actigard is only registered for use on tomato and tobacco. Furthermore, BTH cannot be used on the major crops (wheat, corn, potatoes, soybean, sugarcane, and rice), while isotianil is only effective on rice. Nonetheless, the existence of chemicals boosting crop immunity demonstrates the validity of developing such chemistries as crop protectants. Furthermore, both BTH and Isotianil have been developed without prior knowledge of their receptors. However, the fact, that NPR1 is the receptor for BTH, validates its suitability as a platform to develop new agrochemicals through target-based pharmaceutical-style approaches and raises the perspective that better chemistries with efficacies on a broad range of crops could be designed.

### Advancing NPR1 to a Druggable Target: Differential Scanning Fluorimetry Technology

Drug discovery assays are grouped into two broad categories; cell-based and target-based assays. The former is based on phenotype and includes chemical genetics, which has been used to identify molecules that modulate plant defense response (Knoth et al., 2009; Noutoshi et al., 2012). Target-based assays rely on an isolated protein, typically a receptor, to identify novel ligands, both agonists and antagonists. Given that we have identified a receptor–ligand couple in the form of NPR1-SA, the choice of using a target-based approach for drug discovery seemed logical for us. We have previously shown that NPR1 binds SA using equilibrium dialysis and scintillation proximity assays. Although these methods can be implemented for high throughput assay screens, they are cumbersome and make use of radioligands. In search of a simple, equilibrium based and non-radioactive assay, we opted to focus on thermodynamic methods. These are based on the change of protein thermal stability upon binding to a ligand. In an equilibrium thermodynamics system, a protein population exists in one of two states, the native state (N) and the unfolded state (U) (Adkins, 1983).

**FIGURE 2:**
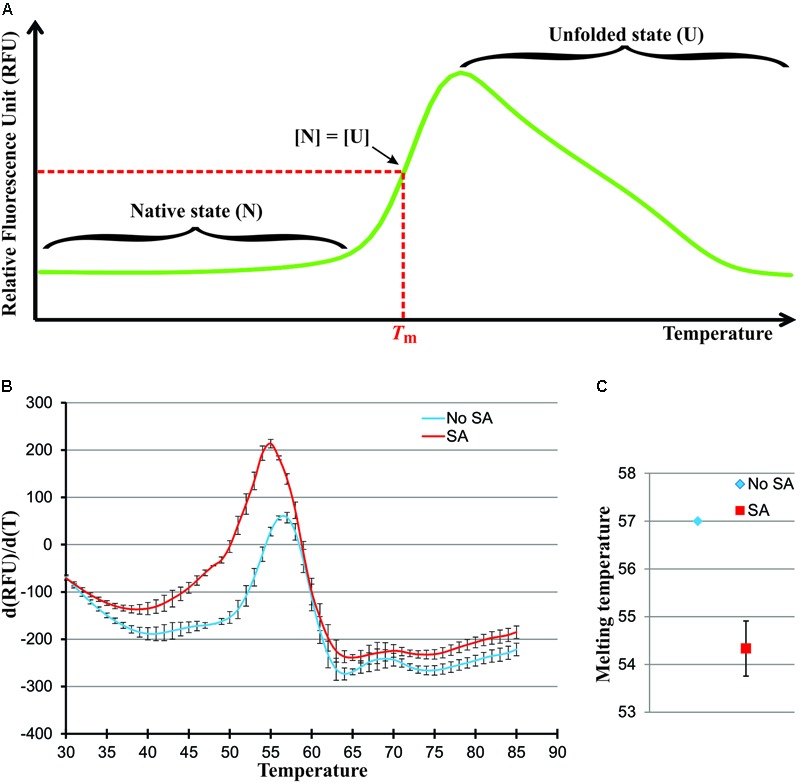
SA destabilize the C-terminus of the NPR1 Δ513. **(A)** Graphic of a typical thermally induced protein unfolding in a DSF experiment. The *T*_m_ is the value at the inflection point, which can be measured more precisely by determining the first derivative. It is typical to observe a loss of fluorescence once proteins are unfolded due to their aggregation. **(B)** First derivation melting curves of Δ513 in the absence (blue) or presence (red) of SA. The *T*_m_ is the value at the maximum, which can be measured more precisely by determining the second derivative. **(C)** Melting temperature, where *T*_m_ is the value of x when *f″(x)* = 0, of Δ513 in the absence (blue) or presence (red) of SA. S.D. calculated from three biological replicates. Note that the S.D. from the No SA control (blue) is smaller than the data point symbol and hence does not show.

If heat is added at a constant rate to this protein population system, the protein molecules will undergo conformational changes gradually shifting the equilibrium toward the unfolded state until every molecule is unfolded. Graphically, this transition from the native to unfolded state, as a function of temperature, appears as depicted in **Figure 2A**. The temperature required to reach the midpoint of this thermal transition, when the concentration of the native and unfolded forms of the protein are equal, is defined as the Melting Temperature (*T*_m_). The *T*_m_ is considered a good indication of protein stability, whereby a higher *T*_m_ is indicative of a higher protein stability. Since ligand binding is known to affect protein stability, a shift in *T*_m_ for a given protein can be observed after ligand binding (Cimmperman et al., 2008). One classic method used to measure the *T*_m_ of protein is differential scanning calorimetry (DSC) (Dassie et al., 2005). In a DSC experiment, the heat absorption of a protein population in a thermally induced transition process is measured. DSC is a very accurate and direct way to measure protein *T*_m_ and other thermodynamic parameters, such as, calorimetric denaturation enthalpy (ΔH). However, this technique is rather low throughput, only one sample can be run at one time and each run typically takes one and half-hour per sample based on our experience. Another method that can be used to monitor protein *T*_m_ is differential scanning fluorimetry (DSF), also known as thermal shift or ThermoFluor (Niesen et al., 2007). A fluorescent dye that non-specifically binds with hydrophobic regions of proteins is used during the course of a thermally induced transition process. The fluorescence of the dye is quenched in aqueous solution but is very high in hydrophobic environments. As protein unfolds, their hydrophobic regions are exposed and bind the dye resulting in an increase in the fluorescence signal. The fluorescence signal emitted by the dye is used to monitor the unfolding process of the protein. In a DSF experiment, the fluorescence intensity can be plotted as a function of temperature. The *T*_m_ values can be calculated simply by determining the maximum of the first derivative. Thermal shift experiments can be done using conventional Real-Time PCR machine and are easily scaled up to 96 or 384 reactions assay. Therefore, it is widely used, in both academia and the pharmaceutical industry, in screens aimed at determining the best conditions for the stability of a given protein and in the early stages of drug discovery (Niesen et al., 2007). Many publications have successfully demonstrated the use of thermal shift in high throughput drug screening (Lo et al., 2004; Vedadi et al., 2006).

The thermal shift assay is therefore an obvious choice for the preliminary screening of small ligands that bind to NPR1. To develop a high-throughput screening assay, we must first ensure that NPR1 can bind SA in a thermal shift assay. We tested the C-terminal S**A**-binding domain of NPR1, Δ513, because of its better folding and higher production yield in *E. coli* protein expression systems. Upon SA binding, the *T*_m_ of Δ513 shifted from 57 to 54.3°C (**Figures 2B,C**). This result shows again the binding of SA to NPR1, bringing the number of assays, that have been used to demonstrate NPR1’s binding to SA, to six (Wu et al., 2012; Manohar et al., 2014). In addition, the data provide new structural insights on the nature of the NPR1-SA complex. The observation, that the *T*_m_ of Δ513 was decreased by 2.6°C upon SA binding, indicates that SA destabilizes Δ513. Given that a protein population exists in two states during a thermal transition process, the native and unfolded states (Eq. 1), there are two possible explanations for why SA destabilizes Δ513. First, SA binds to the unfolded state of Δ513 favoring the unfolding direction of the two-state process, which causes a decrease in *T*_m_.

N_Δ513_ ↔ U_Δ513_         (1)

N_Δ513_ + SA ↔ U_Δ513_⋅SA         (2)

Second, SA binds to the native state which causes the structure of Δ513 to change to a less stable state (Eq. 3) before unfolding (Eq. 4), which results in a decrease in *T*_m_.

N_Δ513_ + SA ↔ Lower-stability_Δ513_⋅SA         (3)

Lower-stability_Δ513_⋅SA ↔ U_Δ513_ + SA         (4).

Finally, once the assay is fine-tuned, the process for turning any assay into a high throughput screen (the scale up) consists in taking the single-tube assay, in our case the method to detect the NPR1-SA interaction, and to perform it simultaneously in a high multiple, for example in a 96-well plate. However, instead of using the same ligand in every well, in our case SA, a chemical library of diversified structures will be used allowing the interrogation of many potential ligands at one time. Chemicals that show a positive signal in an assay are called hits and are then subjected to further rounds of confirmation and analyses.

## Conclusion

Advanced agricultural practices, classical breeding and crop protectants, dominated by fungicides, have been very effective at holding the pendulum away from pathogen prevalence. These “pillars” of modern agriculture have collectively ensured crop productivity and quality for contemporary human civilizations and they will likely continue to play a role in the future. However, the fungicide pillar is under attack and our capacity to hold the pendulum above our head is undermined. We propose to exploit NPR1 as a druggable target to develop “just-in-time” immunomodulating chemistries for crop protection as part of a “holistic” approach to extend the commercial life of effective fungicides and ensure future crop yield security. Plant immunomodulators should show no resistance from pathogens and could be used in an alternate regimen with fungicides or in combination with sublethal doses of fungicides. The SA receptor, NPR1, is a validated target and the DSF technology appears to be promising for the implementation of a target-based pharmaceutical-style high throughput screening platform to develop agonists for next generation crop protectants.

## Methods

### Purification of Recombinant Proteins

Proteins were expressed in *E. coli* as N-terminal fusions to the 6xHIS tag according to standard protocols. Recombinant proteins were purified using HisTrap columns (GE) according to the manufacturer’s protocol. The binding buffer contained 20 mM sodium phosphate at pH 7.2, 40 mM imidazole and 500 mM NaCl. Bound proteins were eluted in the same buffer supplemented with 500 mM imidazole. Proteins were desalted/buffer-exchanged right after purification using MiniTrap columns (GE) according to the manufacturer’s instructions.

### Thermal Shift Assay

One (1) μg of recombinant Δ513 at final concentration of 0.05 μg/μl was used in the thermal shift assays. The reaction buffer consisted of 20 mM sodium phosphate, pH 7.2 and 5× SYPRO Orange dye (Sigma, S5692). The assays were carried with or without 0.03 μM SA. The final reaction volume is 20 μl. Reactions were loaded in Multiplate (Biorad, MLL9601). Thermal shift assays were performed on a CFX96 spectrofluorometric thermal cycler (BioRad) at a scan rate of 1°C/min.

## Author Contributions

XK, CB, and CD wrote the manuscript. XK performed all experiments.

## Conflict of Interest Statement

The authors declare that the research was conducted in the absence of any commercial or financial relationships that could be construed as a potential conflict of interest.
